# Critical review of methodological concerns in ‘Finding the minimum number of retrieved lymph nodes and negative lymph nodes in gastric cancer surgery: a real-world study’

**DOI:** 10.1097/JS9.0000000000001785

**Published:** 2024-06-10

**Authors:** Bing Liu, Shengzhong Wang

**Affiliations:** Department of Gastrointestinal Surgery, Central South University, Xiangya School of Medicine Affiliated Haikou Hospital, Haikou, Hainan Province, People’s Republic of China

*Dear Editor,*

We read with great interest the article titled ‘Finding the minimum number of retrieved lymph nodes and negative lymph nodes in gastric cancer surgery: a real-world study’^1^. The study presents valuable data on the optimal number of retrieved lymph nodes (rLNs) and negative lymph nodes (nLNs) in gastric cancer surgeries. However, we identified several potential issues and inconsistencies that may affect the validity of the conclusions drawn.

First, there is an inconsistency in the reporting of follow-up durations. The Methods section mentions a median follow-up time of 3095 days, while the Results section lists a median follow-up time of 2682 days and an average follow-up time of 3095 days. Consistent reporting of follow-up duration is crucial for the reliability of survival analyses.

Second, the survival benefit associated with higher rLNs and nLNs is highlighted, but the potential for increased surgical morbidity with extensive lymph node dissection is not addressed. This is a critical aspect, as aggressive lymphadenectomy may lead to complications that offset the survival benefits.

Additionally, the study does not account for the potential impact of different surgical techniques (e.g. laparoscopic vs. open surgery) on the number of lymph nodes retrieved and patient outcomes. Given the increasing use of minimally invasive techniques, this factor should be considered.

Furthermore, the survival curves for the two groups cross each other (Figs. 3F, G, 4F, 5C, 6C), yet the *P* values are shown to be less than 0.05. In survival analysis, crossing curves can indicate that the proportional hazards assumption is violated. This assumption is a key requirement for the Cox proportional hazards model, which is often used to calculate the *P*-value in survival analysis. When this assumption is violated, the interpretation of the *P*-value becomes questionable. While the *P*-value is less than 0.05, indicating a statistically significant difference in survival between the two groups, the crossing of curves suggests that the relationship between the groups changes over time. This can complicate the interpretation because the advantage of having more nLNs might not be consistent throughout the entire follow-up period.

Addressing these concerns is crucial for ensuring the accuracy and relevance of the study’s outcomes. We believe that incorporating these adjustments will significantly enhance the robustness of the study’s findings.

## Ethical approval

For the study, ethical approval has been granted by the ethics committee of Central South University Xiangya School of Medicine Affiliated Haikou Hospital.

## Consent

The letter does not involve patients or volunteers.

## Source of funding

Not applicable.

## Author contribution

All authors contributed to the writing of this letter; Shengzhong Wang was responsible for revising the manuscript and responsible for the submission process

## Conflicts of interest disclosure

There are no conflicts of interest.

## Research registration unique identifying number (UIN)

Not registered for the type of study.

## Guarantor

Shengzhong Wang is fully responsible for the work and/or the conduct of the study, has access to the data, and controls the decision to publish.

## Data availability statement

Not applicable.

## Provenance and peer review

Not applicable.
